# 1-Phenyl-2-(1*H*-1,2,4-triazol-1-yl)ethanol

**DOI:** 10.1107/S1600536808017303

**Published:** 2008-06-13

**Authors:** Özden Özel Güven, Hakan Tahtacı, Simon J. Coles, Tuncer Hökelek

**Affiliations:** a Zonguldak Karaelmas University, Department of Chemistry, 67100 Zonguldak, Turkey; b Department of Chemistry, Southampton University, Southampton SO17 1BJ, England; c Hacettepe University, Department of Physics, 06800 Beytepe, Ankara, Turkey

## Abstract

In the title compound, C_10_H_11_N_3_O, the planar five- and six-membered rings are nearly parallel to each other, making a dihedral angle of 2.52 (5)°. Weak inter­molecular C—H⋯O hydrogen bonds link the mol­ecules into centrosymmetric dimers and strong inter­molecular O—H⋯N hydrogen bonds link the dimers into infinite chains along the *b* axis.

## Related literature

For general backgroud, see: Holla *et al.* (1996 ▶); Sengupta *et al.* (1978 ▶); Paulvannan *et al.* (2001 ▶); Sui *et al.* (1998 ▶); Bodey (1992 ▶). For related literature, see: Peeters *et al.* (1979*a* ▶,*b* ▶); Caira *et al.* (2004 ▶); Freer *et al.* (1986 ▶); Peeters *et al.* (1996 ▶).
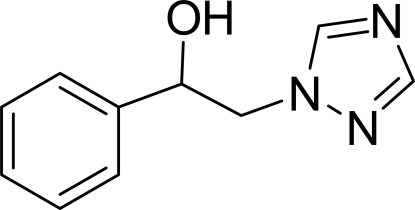


## Experimental

### 

#### Crystal data

C_10_H_11_N_3_O
*M* *_r_* = 189.22
Monoclinic, 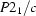

*a* = 11.5356 (2) Å
*b* = 10.1173 (2) Å
*c* = 8.7127 (2) Å
β = 108.581 (1)°
*V* = 963.85 (3) Å^3^
*Z* = 4
Mo *K*α radiation
μ = 0.09 mm^−1^
*T* = 294 (2) K
0.55 × 0.25 × 0.10 mm

#### Data collection

Bruker–Nonius KappaCCD diffractometer
Absorption correction: multi-scan (*SADABS*; Sheldrick, 2007 ▶) *T*_min_ = 0.972, *T*_max_ = 0.989
13352 measured reflections
2208 independent reflections
1647 reflections with *I* > 2σ(*I*)
*R*_int_ = 0.040

#### Refinement

*R*[*F* ^2^ > 2σ(*F* ^2^)] = 0.042
*wR*(*F* ^2^) = 0.115
*S* = 1.03
2208 reflections
171 parameters
All H-atom parameters refined
Δρ_max_ = 0.16 e Å^−3^
Δρ_min_ = −0.21 e Å^−3^

### 

Data collection: *COLLECT* (Hooft, 1998 ▶); cell refinement: *DENZO* (Otwinowski & Minor, 1997 ▶) and *COLLECT*; data reduction: *DENZO* and *COLLECT*; program(s) used to solve structure: *SHELXL97* (Sheldrick, 2008 ▶); program(s) used to refine structure: *SHELXL97* (Sheldrick, 2008 ▶); molecular graphics: *ORTEP-3 for Windows* (Farrugia, 1997 ▶); software used to prepare material for publication: *WinGX* (Farrugia, 1999 ▶).

## Supplementary Material

Crystal structure: contains datablocks I, global. DOI: 10.1107/S1600536808017303/fl2201sup1.cif

Structure factors: contains datablocks I. DOI: 10.1107/S1600536808017303/fl2201Isup2.hkl

Additional supplementary materials:  crystallographic information; 3D view; checkCIF report

## Figures and Tables

**Table 1:** Hydrogen-bond geometry (Å, °)

*D*—H⋯*A*	*D*—H	H⋯*A*	*D*⋯*A*	*D*—H⋯*A*
O—H⋯N2^i^	0.88 (2)	2.00 (2)	2.8645 (17)	166 (2)
C10—H10⋯O^ii^	0.959 (16)	2.566 (16)	3.3198 (17)	135.6 (13)

Symmetry codes: (i) 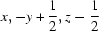; (ii) 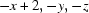.

**Fig. 1.**
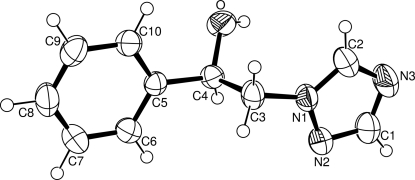
The molecular structure of the title molecule with the atom-numbering scheme. Displacement ellipsoids are drawn at the 50% probability level.

**Fig. 2.**
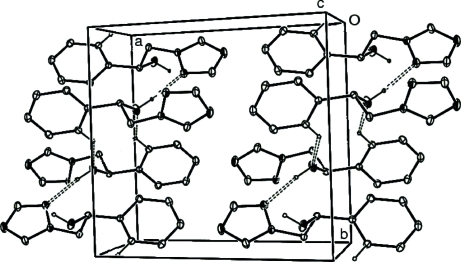
A packing diagram of (I). Hydrogen bonds are shown as dashed lines.

## supplementary crystallographic information

### Comment

Azole derivatives continue to occupy an important place among systemic antifungal drugs. 1,2,4-triazoles are biologically interesting and their chemistry is receiving considerable attention due to their antihypertensive, antifungal and antibacterial properties (Holla *et al.*, 1996; Sengupta *et al.*, 1978; Paulvannan *et al.*, 2001; Sui *et al.*, 1998). The azole antifungals possessing an imidazole or triazole ring (such as miconazole, ketoconazole, fluconazole, econazole and itraconazole) inhibit the synthesis of sterols in fungi by inhibiting cytochrome P-450-dependent 14α-lanosterol demethylase (P-450_14DM_) and prevent cytochrome P-450 activity (Bodey, 1992). The crystal structures of miconazole (Peeters *et al.*, 1997*a*), ketoconazole (Peeters *et al.*, 1979*b*), fluconazole (Caira *et al.*, 2004), econazole (Freer *et al.*, 1986) and itraconazole (Peeters *et al.*, 1996) have already been reported. This paper describes the crystal structure of a 1,2,4-triazole derivative, (I).

In (I) the bond lengths and angles are generally within normal ranges (Fig. 1). The 1,2,4-triazole and benzene rings, A (N1—N3/C1/C2) and B (C5—C10),  are planar  and nearly parallel to each other with a dihedral angle of A/B = 2.52 (5)°. Atoms C3 and C4 are 0.040 (1) Å and -0.046 (1) Å away from the ring planes of A and B, respectively indicating that they are coplanar with the adjacent rings. The N1—C3—C4 [111.53 (10)°] and C3—C4—C5 [109.94 (10)°] bond angles are a little different from each other, while O—C4—C3 [109.53 (11)°] and O—C4—C5 [110.01 (10)°] bond angles are nearly equal. In ring A, the equivalent N1—N2—C1 [102.24 (12)°] and C1—N3—C2 [102.29 (13)°] bond angles are narrowed and approximately equal to one another, while the N3—C2—N1 [111.04 (15)°] and N3—C1—N2 [115.33 (15)°] bond angles are quite different and larger than normal, probably due to the strong intermolecular O—H···N hydrogen bonds (Table 1).

In the crystal packing weak intermolecular C—H···O hydrogen bonds (Table 1) link the molecules into centrosymmetric dimers and strong intermolecular O—H···N hydrogen bonds (Table 1) link the dimers  along the *b* axis (Fig. 2).

### Experimental

For the preparation of the title compound, a mixture of 1-phenyl-2-(1*H*-1,2,4 -triazol-1-yl)ethanone (800 mg, 4.27 mmol) and sodiumborohydride (324 mg, 8.54 mmol) in ethanol (13 ml) was refluxed for 5 h. After evaporation of solvent, the mixture was neutralized with dilute HCl and then refluxed for 30 min. After the mixture was cooled, the solution was alkalinized with NaOH and the precipitate was collected and crystallized from benzene to obtain colorless crystals (yield; 577 mg, 71%).

### Refinement

H atoms were located in difference syntheses and refined isotropically [O—H = 0.88 (2) Å, *U*_iso_(H) = 0.096 (7) Å^2^ and C—H = 0.959 (16)–1.012 (17) Å, *U*_iso_(H) = 0.034 (3)–0.081 (6) Å^2^].

### Crystal data

**Table:**

C_10_H_11_N_3_O	*F*_000_ = 400
*M**_r_* = 189.22	*D*_x_ = 1.304 Mg m^−^^3^
Monoclinic, *P*2_1_/*c*	Mo *K*α radiation λ = 0.71073 Å
Hall symbol: -P 2ybc	Cell parameters from 12727 reflections
*a* = 11.5356 (2) Å	θ = 2.9–27.5º
*b* = 10.1173 (2) Å	µ = 0.09 mm^−^^1^
*c* = 8.7127 (2) Å	*T* = 294 (2) K
β = 108.581 (1)º	Block, colorless
*V* = 963.85 (3) Å^3^	0.55 × 0.25 × 0.10 mm
*Z* = 4	

### Data collection

**Table:**

Bruker–Nonius Roper CCD camera on κ-goniostat diffractometer	2208 independent reflections
Radiation source: Bruker-Nonius FR591 rotating anode	1647 reflections with *I* > 2σ(*I*)
Monochromator: graphite	*R*_int_ = 0.040
Detector resolution: 9.091 pixels mm^-1^	θ_max_ = 27.5º
*T* = 120(2) K	θ_min_ = 3.2º
φ and ω scans	*h* = −14→14
Absorption correction: multi-scan(SADABS; Sheldrick, 2007)	*k* = −13→12
*T*_min_ = 0.972, *T*_max_ = 0.989	*l* = −11→10
13352 measured reflections	

### Refinement

**Table:**

Refinement on *F*^2^	Secondary atom site location: difference Fourier map
Least-squares matrix: full	Hydrogen site location: inferred from neighbouring sites
*R*[*F*^2^ > 2σ(*F*^2^)] = 0.042	All H-atom parameters refined
*wR*(*F*^2^) = 0.115	*w* = 1/[σ^2^(*F*_o_^2^) + (0.0583*P*)^2^ + 0.1287*P*] where *P* = (*F*_o_^2^ + 2*F*_c_^2^)/3
*S* = 1.03	(Δ/σ)_max_ < 0.001
2208 reflections	Δρ_max_ = 0.16 e Å^−^^3^
171 parameters	Δρ_min_ = −0.21 e Å^−^^3^
Primary atom site location: structure-invariant direct methods	Extinction correction: none

### Special details

**Table:**

Geometry. All e.s.d.'s (except the e.s.d. in the dihedral angle between two l.s. planes) are estimated using the full covariance matrix. The cell e.s.d.'s are taken into account individually in the estimation of e.s.d.'s in distances, angles and torsion angles; correlations between e.s.d.'s in cell parameters are only used when they are defined by crystal symmetry. An approximate (isotropic) treatment of cell e.s.d.'s is used for estimating e.s.d.'s involving l.s. planes.
Refinement. Refinement of *F*^2^ against ALL reflections. The weighted *R*-factor *wR* and goodness of fit *S* are based on *F*^2^, conventional *R*-factors *R* are based on *F*, with *F* set to zero for negative *F*^2^. The threshold expression of *F*^2^ > σ(*F*^2^) is used only for calculating *R*-factors(gt) *etc*. and is not relevant to the choice of reflections for refinement. *R*-factors based on *F*^2^ are statistically about twice as large as those based on *F*, and *R*- factors based on ALL data will be even larger.

### Fractional atomic coordinates and isotropic or equivalent isotropic displacement parameters (Å^2^)

**Table:**

	*x*	*y*	*z*	*U*_iso_*/*U*_eq_	
O	0.87872 (10)	0.13609 (12)	0.06628 (11)	0.0588 (3)	
H	0.813 (2)	0.182 (2)	0.013 (3)	0.096 (7)*	
N1	0.72669 (9)	0.10513 (11)	0.27992 (13)	0.0434 (3)	
N2	0.68815 (10)	0.20410 (13)	0.35760 (14)	0.0521 (3)	
N3	0.52850 (11)	0.11273 (16)	0.16751 (19)	0.0727 (4)	
C1	0.56896 (13)	0.20320 (19)	0.2849 (2)	0.0630 (4)	
H1	0.5169 (17)	0.2639 (19)	0.316 (2)	0.081 (6)*	
C2	0.63083 (13)	0.05341 (18)	0.1683 (2)	0.0596 (4)	
H2	0.6363 (16)	−0.0179 (19)	0.098 (2)	0.074 (5)*	
C3	0.85650 (11)	0.07494 (15)	0.32125 (17)	0.0445 (3)	
H31	0.8629 (14)	−0.0199 (17)	0.288 (2)	0.063 (5)*	
H32	0.8938 (14)	0.0825 (15)	0.439 (2)	0.059 (4)*	
C4	0.91941 (11)	0.16584 (13)	0.23363 (14)	0.0376 (3)	
H4	0.8978 (11)	0.2558 (13)	0.2499 (15)	0.034 (3)*	
C5	1.05661 (10)	0.14872 (12)	0.30176 (14)	0.0354 (3)	
C6	1.12425 (12)	0.22987 (15)	0.42648 (16)	0.0480 (3)	
H6	1.0823 (15)	0.2996 (17)	0.467 (2)	0.070 (5)*	
C7	1.24947 (13)	0.21333 (16)	0.49370 (19)	0.0572 (4)	
H7	1.2961 (16)	0.2732 (19)	0.584 (2)	0.079 (5)*	
C8	1.30805 (13)	0.11523 (17)	0.4370 (2)	0.0566 (4)	
H8	1.3973 (17)	0.1018 (18)	0.484 (2)	0.078 (5)*	
C9	1.24185 (13)	0.03446 (15)	0.31286 (19)	0.0529 (4)	
H9	1.2826 (15)	−0.0328 (17)	0.269 (2)	0.065 (5)*	
C10	1.11643 (12)	0.05072 (13)	0.24487 (16)	0.0426 (3)	
H10	1.0711 (14)	−0.0032 (15)	0.1553 (19)	0.055 (4)*	

### Atomic displacement parameters (Å^2^)

**Table:**

	*U*^11^	*U*^22^	*U*^33^	*U*^12^	*U*^13^	*U*^23^
O	0.0489 (6)	0.0914 (8)	0.0316 (5)	0.0183 (6)	0.0065 (4)	0.0033 (5)
N1	0.0314 (5)	0.0530 (6)	0.0427 (6)	−0.0020 (4)	0.0077 (5)	0.0030 (5)
N2	0.0371 (6)	0.0646 (8)	0.0512 (7)	−0.0016 (5)	0.0095 (5)	−0.0048 (6)
N3	0.0349 (6)	0.0949 (11)	0.0780 (10)	−0.0047 (7)	0.0036 (6)	−0.0166 (8)
C1	0.0365 (7)	0.0801 (11)	0.0692 (10)	0.0026 (7)	0.0124 (7)	−0.0067 (9)
C2	0.0399 (8)	0.0711 (10)	0.0607 (9)	−0.0100 (7)	0.0061 (7)	−0.0121 (8)
C3	0.0321 (6)	0.0542 (8)	0.0438 (7)	0.0021 (5)	0.0075 (5)	0.0098 (6)
C4	0.0363 (6)	0.0399 (7)	0.0345 (6)	0.0042 (5)	0.0084 (5)	0.0021 (5)
C5	0.0347 (6)	0.0380 (6)	0.0345 (6)	−0.0004 (5)	0.0122 (5)	0.0041 (5)
C6	0.0440 (7)	0.0515 (8)	0.0465 (7)	−0.0005 (6)	0.0116 (6)	−0.0084 (6)
C7	0.0441 (8)	0.0671 (10)	0.0537 (8)	−0.0100 (7)	0.0060 (6)	−0.0084 (8)
C8	0.0337 (7)	0.0728 (10)	0.0603 (9)	0.0009 (7)	0.0106 (6)	0.0085 (8)
C9	0.0456 (8)	0.0579 (9)	0.0585 (8)	0.0132 (7)	0.0212 (7)	0.0042 (7)
C10	0.0425 (7)	0.0424 (7)	0.0417 (7)	0.0038 (6)	0.0117 (6)	0.0006 (6)

### Geometric parameters (Å, °)

**Table:**

O—C4	1.4144 (15)	C4—H4	0.966 (13)
O—H	0.88 (2)	C5—C4	1.5128 (16)
N1—N2	1.3602 (16)	C5—C6	1.3869 (18)
N1—C2	1.3257 (18)	C5—C10	1.3866 (18)
N1—C3	1.4565 (16)	C6—C7	1.385 (2)
N2—C1	1.3178 (18)	C6—H6	0.982 (18)
N3—C2	1.322 (2)	C7—H7	1.01 (2)
C1—N3	1.341 (2)	C8—C7	1.378 (2)
C1—H1	0.96 (2)	C8—C9	1.376 (2)
C2—H2	0.961 (19)	C8—H8	0.989 (19)
C3—H31	1.012 (17)	C9—H9	0.974 (18)
C3—H32	0.977 (17)	C10—C9	1.3875 (19)
C4—C3	1.5194 (18)	C10—H10	0.959 (16)
			
C4—O—H	112.0 (14)	C3—C4—H4	108.0 (7)
C2—N1—N2	109.10 (12)	C5—C4—C3	109.94 (10)
C2—N1—C3	130.55 (13)	C5—C4—H4	109.7 (7)
N2—N1—C3	120.31 (11)	C6—C5—C4	119.78 (11)
C1—N2—N1	102.24 (12)	C10—C5—C4	121.28 (11)
C2—N3—C1	102.29 (13)	C10—C5—C6	118.92 (12)
N2—C1—N3	115.33 (15)	C5—C6—H6	119.2 (10)
N2—C1—H1	120.7 (11)	C7—C6—C5	120.68 (13)
N3—C1—H1	123.9 (11)	C7—C6—H6	120.1 (10)
N1—C2—H2	123.7 (11)	C6—C7—H7	119.0 (10)
N3—C2—N1	111.04 (15)	C8—C7—C6	120.00 (14)
N3—C2—H2	125.2 (11)	C8—C7—H7	121.0 (10)
N1—C3—C4	111.53 (10)	C7—C8—H8	120.9 (11)
N1—C3—H31	107.0 (9)	C9—C8—C7	119.79 (13)
N1—C3—H32	108.5 (9)	C9—C8—H8	119.3 (11)
C4—C3—H31	109.9 (9)	C8—C9—C10	120.47 (14)
C4—C3—H32	110.9 (9)	C8—C9—H9	120.6 (9)
H31—C3—H32	109.0 (13)	C10—C9—H9	118.9 (9)
O—C4—C3	109.53 (11)	C5—C10—C9	120.14 (13)
O—C4—C5	110.01 (10)	C5—C10—H10	119.5 (9)
O—C4—H4	109.6 (7)	C9—C10—H10	120.3 (9)
			
C2—N1—N2—C1	0.35 (16)	C6—C5—C4—C3	−92.44 (14)
C3—N1—N2—C1	178.19 (12)	C10—C5—C4—O	−34.88 (15)
N2—N1—C2—N3	−0.37 (19)	C10—C5—C4—C3	85.83 (14)
C3—N1—C2—N3	−177.91 (14)	C4—C5—C6—C7	178.13 (12)
C2—N1—C3—C4	94.22 (18)	C10—C5—C6—C7	−0.2 (2)
N2—N1—C3—C4	−83.09 (15)	C6—C5—C10—C9	0.30 (19)
N1—N2—C1—N3	−0.24 (19)	C4—C5—C10—C9	−177.99 (12)
C1—N3—C2—N1	0.2 (2)	C5—C6—C7—C8	−0.2 (2)
N2—C1—N3—C2	0.0 (2)	C9—C8—C7—C6	0.4 (2)
O—C4—C3—N1	−69.27 (14)	C7—C8—C9—C10	−0.3 (2)
C5—C4—C3—N1	169.73 (11)	C5—C10—C9—C8	−0.1 (2)
C6—C5—C4—O	146.85 (12)		

### Hydrogen-bond geometry (Å, °)

**Table:**

*D*—H···*A*	*D*—H	H···*A*	*D*···*A*	*D*—H···*A*
O—H···N2^i^	0.88 (2)	2.00 (2)	2.8645 (17)	166 (2)
C10—H10···O^ii^	0.959 (16)	2.566 (16)	3.3198 (17)	135.6 (13)

Symmetry codes: (i) *x*, −*y*+1/2, *z*−1/2; (ii) −*x*+2, −*y*, −*z*.
